# Enhanced Breakdown Strength and Thermal Conductivity of BN/EP Nanocomposites with Bipolar Nanosecond Pulse DBD Plasma Modified BNNSs

**DOI:** 10.3390/nano9101396

**Published:** 2019-09-30

**Authors:** Yan Mi, Jiaxi Gou, Lulu Liu, Xin Ge, Hui Wan, Quan Liu

**Affiliations:** State Key Laboratory of Power Transmission Equipment & System Security and New Technology, School of Electrical Engineering, Chongqing University, Chongqing 400044, China; cqugjx@163.com (J.G.);

**Keywords:** bipolar nanosecond pulse, low temperature plasma, hydroxylation modification, BN nanosheets, nanocomposites, breakdown strength, thermal conductivity

## Abstract

Filling epoxy resin (EP) with boron nitride (BN) nanosheets (BNNSs) can effectively improve the thermal conductivity of BN/EP nanocomposites. However, due to the few hydroxyl groups on the surface of BNNSs, silane coupling agent (SCA) cannot effectively modify BNNSs. The agglomeration of BNNSs is severe, which significantly reduces the AC breakdown strength of the composites. Therefore, this paper uses atmospheric pressure bipolar nanosecond pulse dielectric barrier discharge (DBD) Ar+H_2_O low temperature plasma to hydroxylate BNNSs to improve the AC breakdown strength and thermal conductivity of the composites. X-ray photoelectron spectroscopy (XPS) shows that the hydroxyl content of the BNNSs surface increases nearly two fold after plasma modification. Fourier transform infrared spectroscopy (FTIR) and thermogravimetric analysis (TGA) show that plasma modification enhances the dehydration condensation reaction of BNNSs with SCA, and the coating amount of SCA on the BNNSs surface increases by 45%. The breakdown test shows that the AC breakdown strength of the composites after plasma modification is improved under different filling contents. With the filling content of BNNSs increasing from 10% to 20%, the composites can maintain a certain insulation strength. Meanwhile, the thermal conductivity of the composites increases by 67% as the filling content increases from 10% (SCA treated) to 20% (plasma and SCA treated). Therefore, the plasma hydroxylation modification method used in this paper can provide a basis for the preparation of high thermal conductivity insulating materials.

## 1. Introduction

Breakdown strength and thermal conductivity of insulating materials are important factors affecting the safe and stable operation of electrical equipments. With the development of UHV transmission technology, higher and higher requirements for the performance of insulating materials are put forward. Epoxy resin has good insulation properties and is often used in the electronics and power industries, such as power electronics packaging materials, dry-type transformers, bushings and so on. However, the thermal conductivity of epoxy resin is low, and the heat generated during operation of a device cannot be dissipated in time, resulting in a decrease in the dielectric strength and insulation life of epoxy resin [1,2]. At present, a common method to improve the thermal conductivity of epoxy resin is to add thermal conductive insulating nanoparticles, such as Al_2_O_3_, Si_3_N_4_, AlN, and BN, to the epoxy resin [3,4,5]. Studies have shown that BN has a higher thermal conductivity than other particles, and it has a better effect on the thermal conductivity of epoxy resin. The higher the content of BN, the higher the thermal conductivity of the epoxy resin composite [6,7]. However, when the BN content exceeds a certain amount, serious agglomeration will occur, which will lead to a decrease in the AC breakdown strength of the composite, thus limiting the improvement in the thermal conductivity and practical application of epoxy resin composites [8,9]. Therefore, it is important to improve the breakdown strength of high thermal conductivity nanocomposites.

Further research shows that improving the dispersion of nanoparticles in matrix materials is an effective method to improve the insulation properties of nanocomposites, and this is also a research hotspot and challenge for nanomaterials [10,11,12]. At present, the main method to improve the dispersion of nanoparticles is surface modification of nanoparticles, and silane coupling agent (SCA) is a commonly used surface modifier [13]. However, the surface of BN has a small content of -OH groups [14], and the bonding degree between BN and SCA is low, so it is necessary to hydroxylate the surface first. The methods for surface hydroxylation modification of BN mainly include high temperature annealing [15], NaOH heat treatment [16], H_2_O_2_ heat treatment [17], and concentrated mixed acid treatment [18]. However, these methods take a long time, and the strong oxidizing chemical reagents used in these methods are not environmentally friendly.

In recent years, some scholars have begun to modify nanoparticles by plasma. Hydroxyl groups are bonded on the surface of nanoparticles by the collisions of high-energy particles in plasma, and this method has the advantages of high efficiency and environmental friendliness. Guangning Wu et al. [19] modified nano-Al_2_O_3_ with atmospheric pressure air plasma excited by high frequency AC power, and effectively increased the hydroxyl content on the surface of Al_2_O_3_. Yeongseon Kim et al. [14] used atmospheric pressure RF plasma to modify BN and successfully introduced hydroxyl groups on the BN surface, which enhanced the binding degree of BN and SCA. Thus, a high-frequency AC source is currently used to hydroxylate nanoparticles. Some studies show that nanosecond pulse discharge can produce more uniform plasma and has a higher energy efficiency than high-frequency AC power because nanosecond pulse power has a shorter rising edge than AC power, which can trigger overvoltage breakdown and enhance the ionization and electronic excitation processes [20,21,22]. Moreover, pulse discharge plasma has a lower temperature and less influence on the material [23].

In addition to the above advantages, bipolar nanosecond pulse dielectric barrier discharge (DBD) has a reverse electric field generated by the charge accumulated on the surface of the dielectric after the last pulse discharge, and this field is in the same direction as the electric field generated by the next pulse. The electric field is enhanced after superposition, thereby promoting the development of discharge and the generation of active particles. Yunfei Liu et al. [24] used atmospheric pressure bipolar nanosecond pulse discharge to generate plasma in air to modify the surface of PET films. Experiments showed that uniform plasma can be generated when the air gap is less than 1.2 mm; the surface of the modified film had no obvious morphological change, and oxygen-containing polar groups were successfully introduced. Dezheng Yang et al. [25] studied the atmospheric pressure air DBD emission spectra driven by different polarity pulses. The results showed that the N_2_ (C_3_П_u_ → B_3_П_g_) spectral emission intensity produced by bipolar pulse discharge was approximately 4–5 times that of unipolar pulse discharge, which indicated that bipolar pulse discharge was more conducive to exciting active substances.

In view of the current problems existing in BN/EP composites and the advantages of plasma modification by bipolar nanosecond pulse discharge, this paper uses atmospheric pressure bipolar nanosecond pulse DBD Ar+H_2_O low temperature plasma for hydroxylation modification of BNNSs to enhance the combination of BNNSs and SCA, increase the coating amount of SCA, and improve the AC breakdown strength and thermal conductivity of BN/EP nanocomposites, which provides a basis for the preparation of high thermal conductivity insulating materials for power systems.

## 2. Materials and Methods

### 2.1. Materials

**Figure 1:**
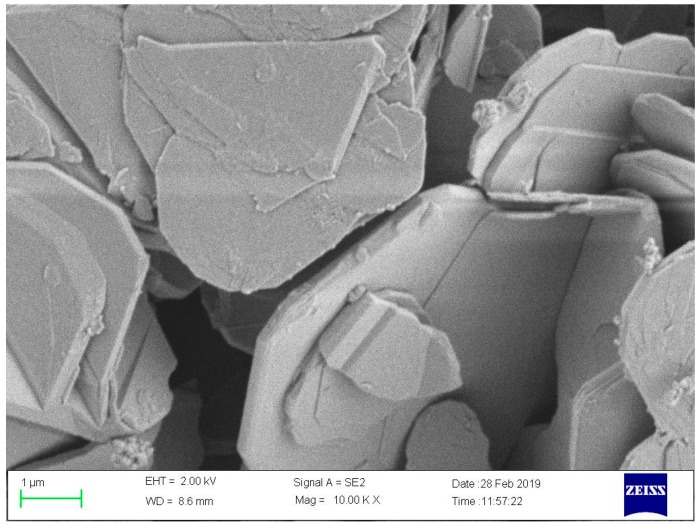
SEM image of pristine epoxy resin (EP) with BN nanosheets (BNNSs).

The average particle size of the hexagonal BNNSs was 50 nm, and the BNNSs were purchased from Chaowei Nanotechnology Co., Ltd. (Shanghai, China). The scanning electron microscopy (SEM) image is shown in Figure 1. Ar (purity ≥ 99.999%) was purchased from Chaoyang Gas Co., Ltd. (Chongqing, China). The SCA KH560 and 0.1 M oxalic acid titration solution were purchased from Aladdin Biochemical Technology Co., Ltd. (Shanghai, China). Bisphenol A epoxy resin E51, methyl tetrahydrophthalic anhydride (MeTHPA) curing agent HKR-0719, and the accelerant DMP-30 were purchased from Huakai Resin Co., Ltd. (Jining, Shandong, China). Anhydrous ethanol was purchased from Cologne Chemicals Co., Ltd. (Chengdu, Sichuan, China). Pure water was purchased from Chuandong Chemical (Group) Co., Ltd. (Chongqing, China).

### 2.2. Plasma Modification of Epoxy Resin (EP) with BN Nanosheets (BNNSs)

**Figure 2:**
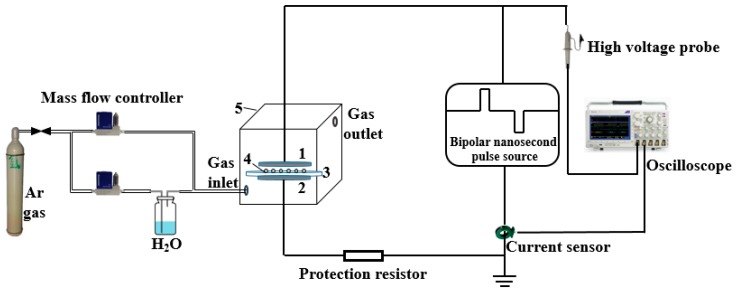
Schematic diagram of plasma modification of BNNSs. 1: High voltage electrode, 2: ground electrode, 3: silica glass, 4: BNNSs, 5: glass box.

**Figure 3:**
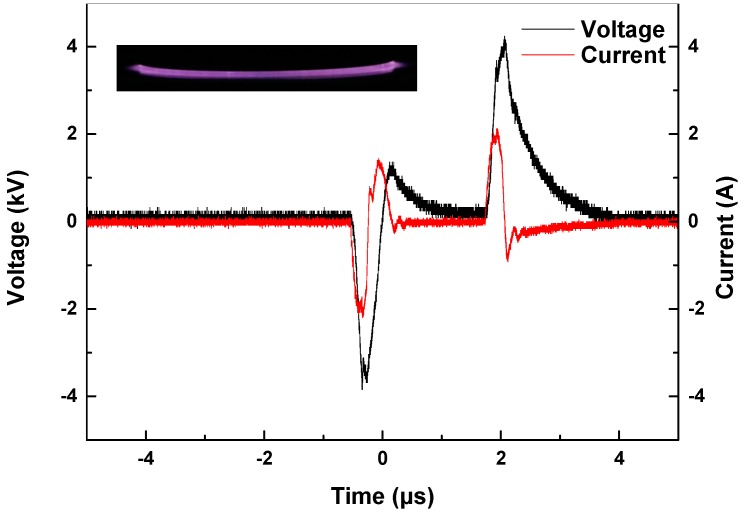
Voltage and current waveforms of dielectric barrier discharge (DBD) and image of plasma.

A schematic diagram of the plasma modification is shown in Figure 2. The power supply is a laboratory-made bipolar nanosecond pulse generator [26]. The electrode plates are stainless-steel circular electrodes with a diameter of 100 mm. The barrier dielectric is a circular quartz glass with a diameter of 110 mm and a thickness of 1 mm. A 500 Ω noninductive resistor is also connected in series to DBD as a protective resistor. The whole DBD device is placed in a transparent glass box, and two gas channels are placed the box. One channel is directly connected to high-purity Ar, while through the other channel, Ar is passed into a gas-washing bottle containing pure water. The flow ratio of the two is 4:1 and the total flow rate is 2 L/min. The flow rate is controlled by a D07-7 gas mass flow controller (Qixing Huachuang Electronics Co., Ltd., Beijing, China). In the experiment, 0.5 g BNNSs is spread on the quartz glass and then placed on the ground electrode plate. The distance between the plates is 2 mm. The glass box is placed over the DBD device and filled with Ar and H_2_O mixed gas for 3 min. Then the generator is activated to produce plasma for 30 s. BNNSs are removed, stirred well and placed into the glass box for another 30 s treatment. This process is repeated three times to complete the hydroxylation modification. The voltage and current waveforms of DBD are measured by a high voltage probe P6015A (Tektronix. Inc., Beaverton, OR, USA), Pearson current sensor 2877 (Pearson Electronics. Inc., Palo Alto, CA, USA) and an oscilloscope DPO 4054 (Tektronix. Inc., Beaverton, OR, USA). The waveforms are shown in Figure 3. The voltage amplitude is 4 kV, the pulse width is 300 ns, the frequency is 1 kHz, and the positive and negative pulse interval is 2 μs. The current exhibits the typical characteristic of pulse DBD. There is a pulse current at both the rising and falling edges, and the former is larger than the latter. This difference is because the charge accumulated on the dielectrics at the beginning of discharge is still very small, so the previous current is mainly affected by the applied electric field, and the latter current is generated by the inverse electric field generated by the charge accumulated on the dielectrics minus the applied electric field [21]. The plasma image is taken by a Canon EOS 750D camera (Canon Inc., Tokyo, Japan) with an exposure time of 100 ms, an aperture value of f/10, and an ISO speed of 6400. The image is shown in Figure 3. The plasma fills the entire air gap uniformly, which is advantageous for surface modification of BNNSs.

### 2.3. SCA Treated BNNSs

**Figure 4:**
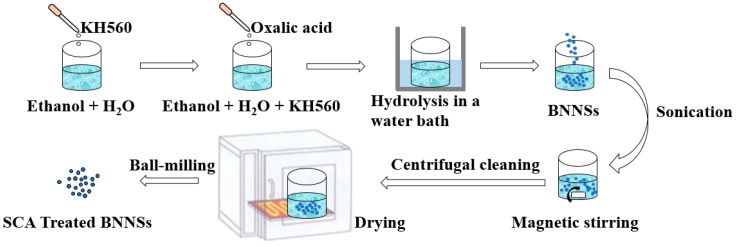
Silane coupling agent (SCA) treatment process.

The treatment process of the SCA is shown in Figure 4. A certain amount of SCA KH560 is incorporated into a mixed solution of absolute ethanol and pure water. The mass ratio of SCA, ethanol and pure water is 1:144:16. The amount of SCA is estimated by the following formula [27]:

(1) $$m_{0}=m.s/s_{0},$$

where *m*_0_ is the amount of SCA, *m* is the mass of the nanoparticles, *s* is the specific surface area of the nanoparticles (34.318 m^2^·g^−1^), *s*_0_ is the minimum coating area of SCA (322 m^2^·g^−1^). In general, the actual amount is less than the calculated amount. Therefore, the amount of SCA is 10% of the mass of the nanoparticles. The pH of the mixed solution is adjusted to 4 with oxalic acid, and then hydrolyzed in a water bath at 40 °C for 2 h. Quantitative BNNSs are then added to the mixed solution and sonicated for 30 min. The solution is stirred with a magnetic stirrer at 800 r/min for 2 h. Then, the suspension is centrifuged for 3 times with absolute ethanol for 5 min each time at a rotation speed of 3000 r/min to remove SCA and other impurities without grafting. The product is then dried in a vacuum oven at 60 °C for 12 h. Finally, the dried BNNSs are ground into powder by a planetary ball mill.

### 2.4. Preparation of the Epoxy Composites

**Figure 5:**
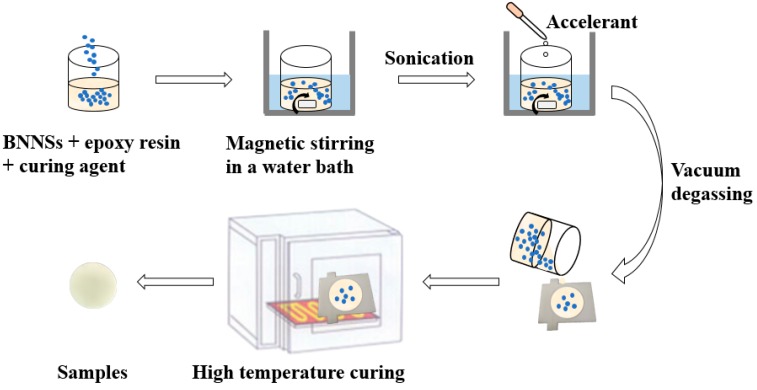
Preparation process of the epoxy composites.

The sample preparation process of the BN/EP nanocomposites is shown in Figure 5. A certain proportion of epoxy resin, curing agent and BNNSs are magnetically stirred in a water bath at 70 °C for 1 h at a speed of 800 r/min. Then, the solution is ultrasonically dispersed in a water bath at 70 °C for 30 min. A certain amount of accelerant (the mass ratio of epoxy resin, curing agent and accelerant is 10:8:0.2) is added, and magnetic stirring is continued for 15 min. The resulting solution is then placed in an oven and degassed under vacuum at 60 °C for 30 min. Finally, the mixture is poured into a mold, and cured in an oven at 90 °C for 2 h and then at 110 °C for 2 h.

### 2.5. Characterization

The elements and hydroxyl groups on the surface of the BNNSs before and after plasma modification were analyzed by X-ray photoelectron spectroscopy (XPS, ESCALAB 250Xi, Thermo Fisher Scientific Co., Ltd., MA, USA). The binding energy was calibrated with reference to the C1s peak of 284.8 eV. Fourier transform infrared spectroscopy (FTIR, Nicolet iS50, Thermo Fisher Scientific Co., Ltd., MA, USA) was used to test whether SCA was grafted onto the BNNSs surface, and the spectrum was recorded from 4000 cm^−1^ to 500 cm^−1^ with a resolution of 4 cm^−1^. TGA of pristine BNNSs and SCA treated BNNSs was carried out using a thermogravimetric differential thermal analyzer (TGA/DSC1/1600LF, METTLER TOLEDO Group, Zurich, Switzerland) at a heating rate of 10 °C/min under a nitrogen atmosphere from 25 °C to 800 °C to evaluate the amount of coating of SCA on BNNSs. The HCDJC-100kV voltage breakdown tester (Huace Instrument Co., Ltd., Beijing, China) was used to test the AC breakdown strength of the BN/EP nanocomposites at different contents. The electrodes are stainless-steel ball electrodes with a diameter of 20 mm. The thickness of the samples is 1mm, and the diameter is 40 mm. To prevent flashover, the sample is immersed in silicone oil. It is uniformly pressurized at a speed of 2 kV/s until breakdown. The thermal conductivity of the nanocomposites was measured by a laser thermal conductivity meter (LFA467HT, Netzsch. Ltd., Selb, Germany).

### 2.6. Statistical Analysis

The experimental data were statistically analyzed using OriginPro software, and the breakdown strength data were fitted using MATLAB software. One-way ANOVA was used to assess statistically significant differences in the experimental data (*p* < 0.05 was considered statistically significant).

## 3. Results and Discussion

### 3.1. X-Ray Photoelectron Spectroscopy (XPS)

**Figure 6:**
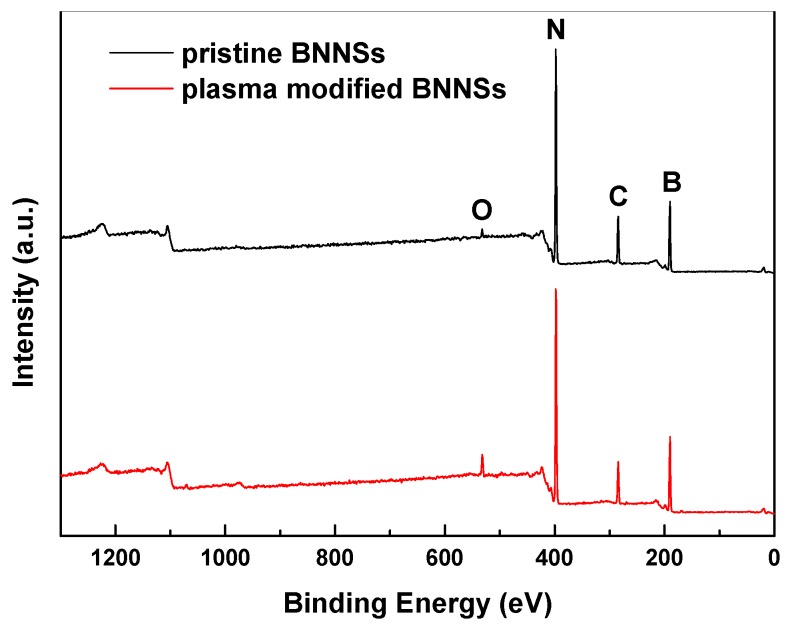
XPS survey spectra of pristine BNNSs and plasma modified BNNSs.

**Figure 7:**
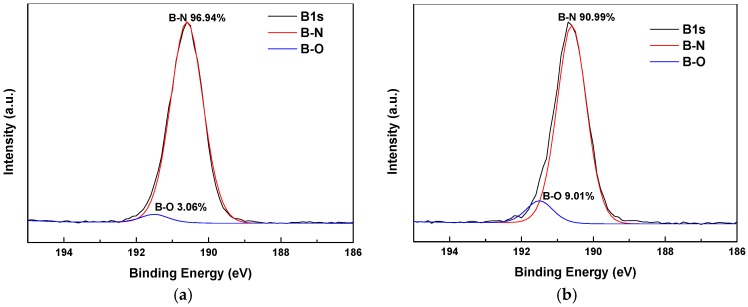
X-ray photoelectron spectroscopy (XPS) spectra of B1s of (**a**) pristine BNNSs, and (**b**) plasma modified BNNSs.

The XPS spectra of BNNSs before and after plasma modification are shown in Figure 6. It can be seen that the BNNSs mainly have B, N, C and O on the surface, and the O peak of the BNNSs is obviously enhanced after plasma modification. To further analyze the effect of plasma hydroxylation modification, the peak of B1s was fitted, and the peak results are shown in Figure 7. The peak at 190.6 eV represents the B-N bond, and the peak at 191.5 eV represents the B-O bond, which forms –OH groups on the BNNSs [28]. Therefore, the effect of the hydroxylation modification can be measured by the content of the B-O bond. After plasma modification, the content of the B-O bond increased from 3.06% to 9.01%, indicating that the plasma modification significantly increased the hydroxyl content of the BNNSs surface. 

This increase is mainly due to the physical and chemical reactions initiated by the collision of high-energy particles in the plasma. First, the high-energy electrons generated by the discharge collide with the ground-state argon atoms, as the reactions reported in the article [29].

Since the ionization energy of argon is relatively high, in addition to direct ionization, it is excited to generate excited state argon. Then excited state argon atoms are ionized to generate more charged particles or returns to the ground state to release photons, further promoting the development of discharge. High-energy particles, such as electrons, excited state argon atoms and photons, have higher energy to break the B-N bond and create active sites on the B atom, which provides a basis for hydroxyl groups bonding.

Hydroxyl groups are mainly produced by ionization decomposition of water. The main reactions are reported in the article [30].

There are two main ways for water to decompose to produce hydroxyl groups. One is the direct collision between electrons and water molecules, and the other is the collision between excited argon atoms and water molecules. The generated hydroxyl group has excess electrons, and can interact with the electron-deficient B atom to form the covalent bond B-OH through Lewis acid-base interactions [28], thereby increasing the hydroxyl content of the BNNSs.

### 3.2. Fourier Transform Infrared Spectroscopy (FTIR)

**Figure 8:**
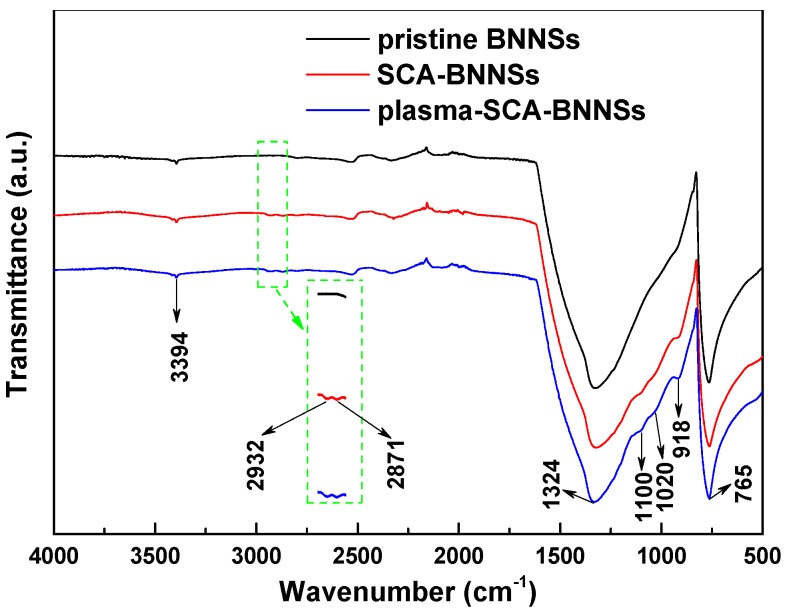
Fourier transform infrared spectroscopy (FTIR) spectra of pristine BNNSs, SCA treated BNNSs (SCA-BNNSs), and plasma and SCA treated BNNSs (plasma-SCA-BNNSs).

**Figure 9:**
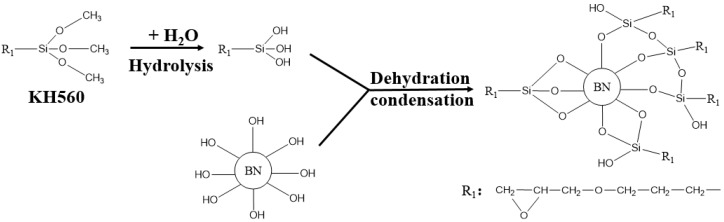
Schematic diagram of the reaction between SCA and BNNSs.

Figure 8 is the Fourier transform infrared spectra of pristine BNNSs, SCA treated BNNSs, plasma and SCA treated BNNSs. All three particles have three absorption peaks at 3394, 1324, and 765 cm^−1^, which represent the -OH vibration peak, the B-N stretching vibration and bending vibration peak, respectively [5]. After SCA treatment, weak -CH2- antisymmetric and symmetric stretching vibration peaks appear at 2932 and 2871 cm^−1^, which represent the carbon chain in the organic functional group of SCA [31]. A Si-O-Si stretching vibration peak appears at 1100 cm^−1^, which is formed by the dehydration condensation reaction of Si-OH in the SCA. The Si-O-C, which is a hydrolyzable functional group of SCA, stretching vibration peak appears at 1020 cm^−1^, indicating that some SCA is not sufficiently hydrolyzed. 918 cm^−1^ is the vibration peak of B-O-Si [32], which is formed by the dehydration condensation reaction between B-OH on the surface of BNNSs and Si-OH of SCA. Moreover, the BNNSs treated with plasma and SCA have a stronger B-O-Si vibration peak than those treated with SCA only. This difference is because the plasma modification increases the hydroxyl content of the BNNSs, thereby enhancing the degree of binding of the BNNSs to SCA. A schematic diagram of the reaction between SCA and BNNSs is shown in Figure 9.

### 3.3. Thermogravimetric Analysis (TGA)

**Figure 10:**
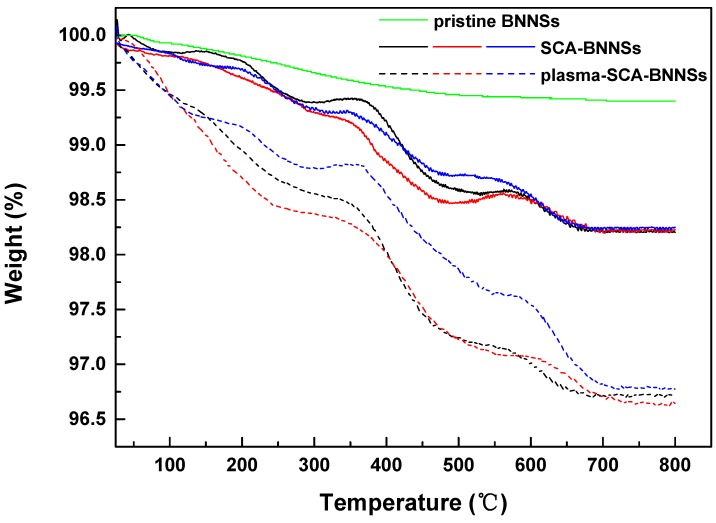
Thermal degradation of pristine BNNSs, SCA treated BNNSs (SCA-BNNSs), and plasma and SCA treated BNNSs (plasma-SCA-BNNSs). The interval between each treatment and test is less than 1 h.

**Figure 11:**
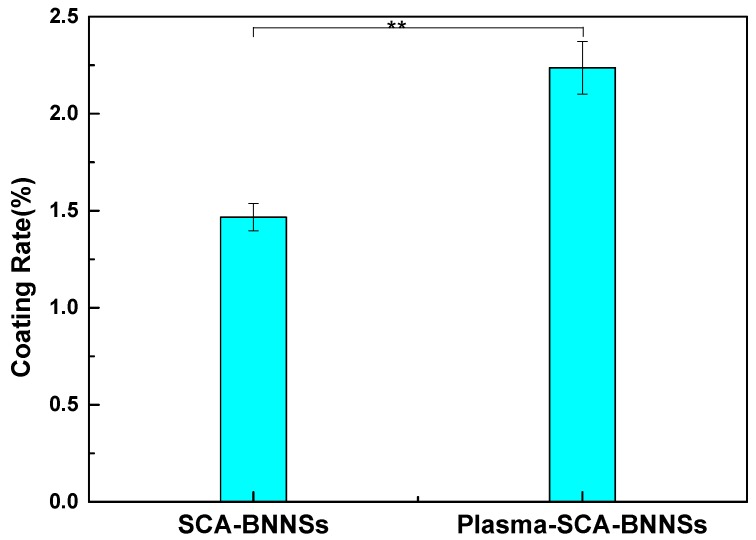
Coating rate of SCA treated BNNSs (SCA-BNNSs), and plasma and SCA treated BNNSs (plasma-SCA-BNNSs). ** *p* < 0.01. The interval between each treatment and test is less than 1 h.

Figure 10 shows the thermogravimetric curves of pristine BNNSs, SCA treated BNNSs, and plasma and SCA treated BNNSs. It can be seen that pristine BNNSs are very stable, and their weight does not change substantially as the temperature increases. The BNNSs treated by the two methods show different degrees of weight loss. The weight loss at approximately 200 °C is most likely caused by the thermal desorption of water [32]. It can be seen that plasma and SCA treated BNNSs have more dehydration weight loss, which may be because some of the hydroxyl groups on the surface of the BNNSs do not react with SCA, and there are more redundant hydroxyl groups after plasma modification that adsorb more water. In addition, a portion of Si-OH does not react with B-OH and may adsorb more water. The weight loss from 200 °C to 800 °C is caused by the decomposition of SCA; thus, the coating rate of SCA can be estimated by subtracting the mass fractions of 200 °C and 800 °C. To estimate more accurately, the TGA tests of SCA-BNNSs and plasma-SCA-BNNSs were carried out 3 times, respectively. The interval between each treatment and test is less than 1 h. The results are shown in Table 1 and Figure 11. After the plasma modification, the average SCA coating rate increases from 1.47% to 2.24%, which is 52.4% higher and consistent with the experimental results in Section 3.2.

### 3.4. Breakdown Strength

In the breakdown test, 10 samples were prepared for each kind of composites, and then analyzed using two-parameter Weibull distribution [33]:

(2) $$P=1-\exp\left[-{{\left(\frac{E}{\alpha }\right)}^{\beta }}^{}\right],$$

where *P* is the breakdown probability when the field strength is *E*, *E* is the breakdown field strength of the test, *α* is the size parameter or characteristic breakdown field strength, indicating the field strength value when the breakdown probability is 63.2%, *β* is the shape parameter, indicating the dispersion of the experimental data; the smaller the value, the greater the dispersion. For the breakdown field strength measured for each sample, the breakdown probability can be calculated using the following formula:

(3) $$P_{i}=\frac{i-0.5}{n+0.25}\times 100\%,$$

where *i* represents the ordinal number of the *E* value in ascending order, and *n* represents the total number of test samples for each material. Perform two logarithmic operations on Equation (2), and it can be transformed into a linear Equation:

(4) $$\mathrm{lg}\left(\ln\left(\frac{1}{1-P}\right)\right)=\beta \mathrm{lg}E-\beta \mathrm{lg}\alpha ,$$

Therefore, lg(ln(1/(1−*P*))) can be linearly fitted to lg*E* to calculate the characteristic breakdown strength *α*.

**Figure 12:**
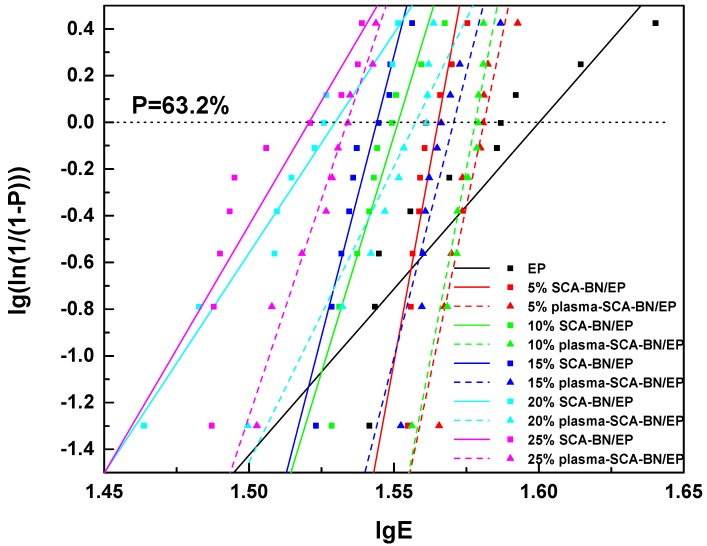
Weibull plots of the breakdown strength of epoxy resin (EP) and BN/EP nanocomposites with SCA treated BNNSs (SCA-BN/EP) and plasma and SCA treated BNNSs (plasma-SCA-BN/EP) at different contents (5%, 10%, 15%, 20%, 25%).

**Figure 13:**
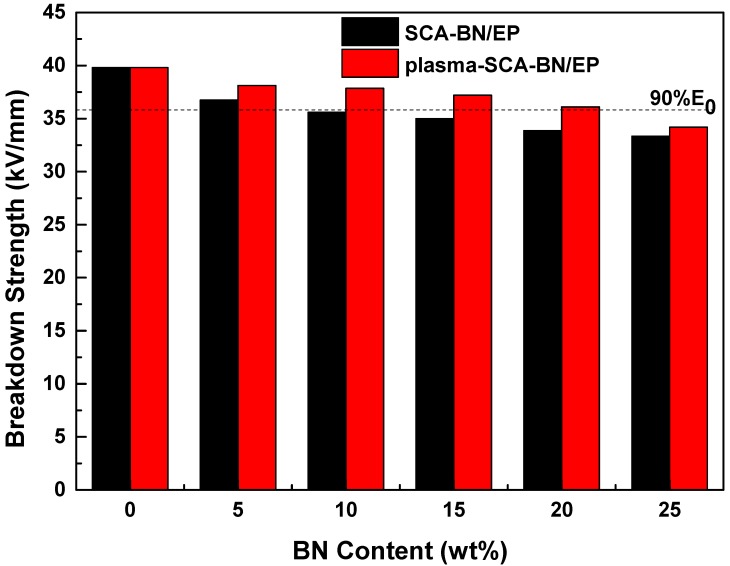
Breakdown strength of the EP and BN/EP nanocomposites with SCA treated BNNSs (SCA-BN/EP) and plasma and SCA treated BNNSs (plasma-SCA-BN/EP) at different contents.

**Figure 14:**
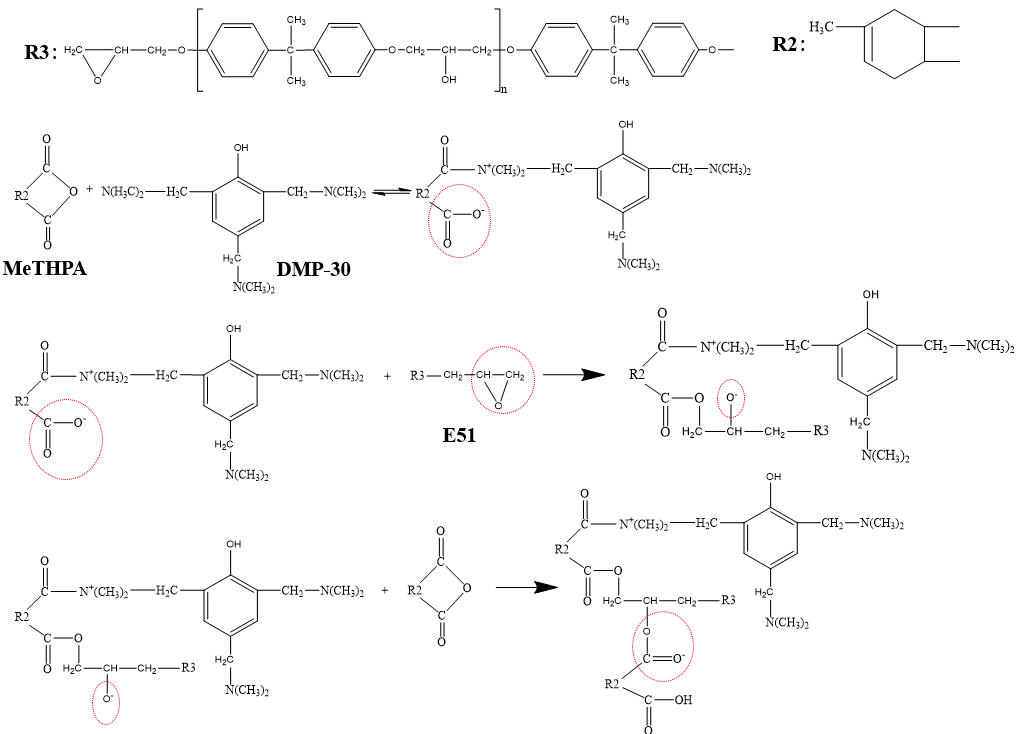
Schematic diagram of curing reaction of epoxy resin.

Figure 12 is the Weibull distribution of the AC breakdown strength of the composites at different contents, the parameters of the Weibull analysis are shown in Table 2. *β* is greater than 14, even close to 70, indicating that the dispersion of experimental data of breakdown strength is small. *R*^2^ is greater than 0.8, indicating that the degree of fit is better. Figure 13 is the characteristic breakdown strength of the composites at different contents. The breakdown strength of both kinds of composites decreases with increasing BNNSs content, but the plasma and SCA treated samples have higher strength than the samples treated only with SCA. This difference may be due to the increase in the hydroxyl groups on the surface of the BNNSs after plasma modification, which improves the coating rate of SCA. The curing reaction of epoxy resin is shown in Figure 14. Since the KH560 SCA has an epoxy group, it can participate in the curing reaction of the epoxy resin, thereby enhancing the degree of bonding between BNNSs and EP matrix. The dispersion of BNNSs in the epoxy resin is improved and the area of the interface bonding region increases. Therefore, there are more deep traps introduced in the nanocomposites [34,35]. On the one hand, deep traps reduce the mobility and average free path of carriers. On the other hand, the trapped carriers near the electrode form space charges of the same polarity, weakening the electric field near the electrode and suppressing the charge injection. The polymer chain fracture becomes difficult and the breakdown strength increases [36]. If 90% of the breakdown strength of pure epoxy resin is acceptable for insulation, the content of BNNSs can increase from approximately 10% to 20% after plasma modification, which will help to improve the thermal conductivity of the composites.

### 3.5. Thermal Conductivity

**Figure 15:**
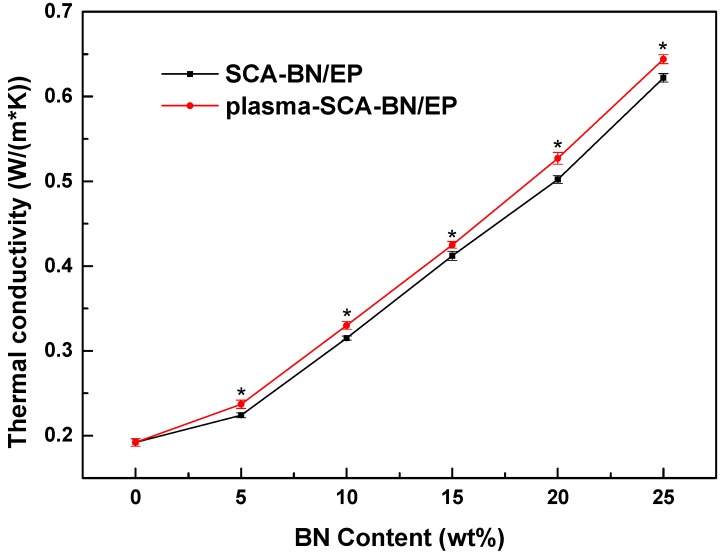
Thermal conductivity of BN/EP nanocomposites with SCA treated BNNSs (SCA-BN/EP) and plasma and SCA treated BNNSs (plasma-SCA-BN/EP) at different contents. * *p* < 0.05.

Figure 15 shows the thermal conductivity of the two kinds of composites at different contents. The values of three samples for each material were averaged and analyzed for significance. The higher the BNNSs content, the higher the thermal conductivity. Moreover, the thermal conductivity of the plasma and SCA treated samples is higher than that of samples treated only with SCA, which may be because the plasma modification increases the hydroxyl content of the BNNSs, thereby increasing the coating rate of SCA. Increasing the coating enhances the interface bonding ability of the BNNSs and epoxy resin, improving the compatibility of the two materials, reducing phonon scattering and interface thermal resistance, and improving the thermal conductivity [37,38]. In addition, combined with the breakdown strength in Section 3.4, when the BNNSs content is increased from 10% (SCA treated) to 20% (plasma and SCA treated), the thermal conductivity of the composites will increase by 67%.

## 4. Conclusions

To simultaneously improve the AC breakdown strength and thermal conductivity of BN/EP nanocomposites, the hydroxylation of BNNSs was carried out by atmospheric pressure bipolar nanosecond pulse DBD Ar+H_2_O low temperature plasma. XPS results indicate that plasma modification significantly increases the hydroxyl content of the BNNSs. The FTIR and TGA results show that more hydroxyl groups and SCA are combined after plasma modification, which improves the coating rate of SCA on the BNNSs surface. The breakdown test and thermal conductivity results show that the plasma and SCA treated nanocomposites have higher breakdown strength and thermal conductivity than those treated with only SCA, although the increased amplitude is not very large. At the same breakdown strength (90% of the breakdown strength of pure epoxy resin), the BNNSs content can increase to 20% after plasma modification, whereby the thermal conductivity of the composites will increase by 67%. Therefore, this paper provides a simple, efficient and environmentally friendly plasma hydroxylation modification method, which also provides effective guidance for the preparation of high thermal conductivity insulating composites. Of course, this method needs further improvement to achieve higher breakdown strength for practical applications.

## Figures and Tables

**Table 1:** Coating rate of SCA treated BNNSs (SCA-BNNSs), and plasma and SCA treated BNNSs (plasma-SCA-BNNSs).

Sample Number	SCA-BNNSs	Plasma-SCA-BNNSs
1	1.45%	2.39%
2	1.56%	2.26%
3	1.39%	2.06%
Average	1.47%	2.24%

**Table 2:** Parameters of the Weibull analysis.

Samples	α (kV/mm)	β	R^2^
EP	39.81	14.23	0.8134
5% SCA-BN/EP	36.75	67.53	0.8493
5% plasma-SCA-BN/EP	38.11	58.70	0.8690
10% SCA-BN/EP	35.60	40.55	0.8885
10% plasma-SCA-BN/EP	37.85	65.46	0.9345
15% SCA-BN/EP	35.00	48.10	0.9172
15% plasma-SCA-BN/EP	37.21	48.93	0.8715
20% SCA-BN/EP	33.86	18.84	0.9612
20% plasma-SCA-BN/EP	36.10	25.31	0.9174
25% SCA-BN/EP	33.33	21.24	0.8079
25% plasma-SCA-BN/EP	34.19	37.20	0.9622
